# Supplementary material for: A Non-Randomized Trial Investigating the Impact of Brown Rice Consumption on Gut Microbiota, Attention, and Short-Term Working Memory in Thai School-Aged Children
Source: Nutrients. 2022 Dec 5;14(23):5176. doi: 10.3390/nu14235176 (PMC9738792; doi:10.3390/nu14235176)
Supplement: Supplementary file 1 [file nutrients-14-05176-s001.zip › Supplementary figures.pdf]

Supplementary figures

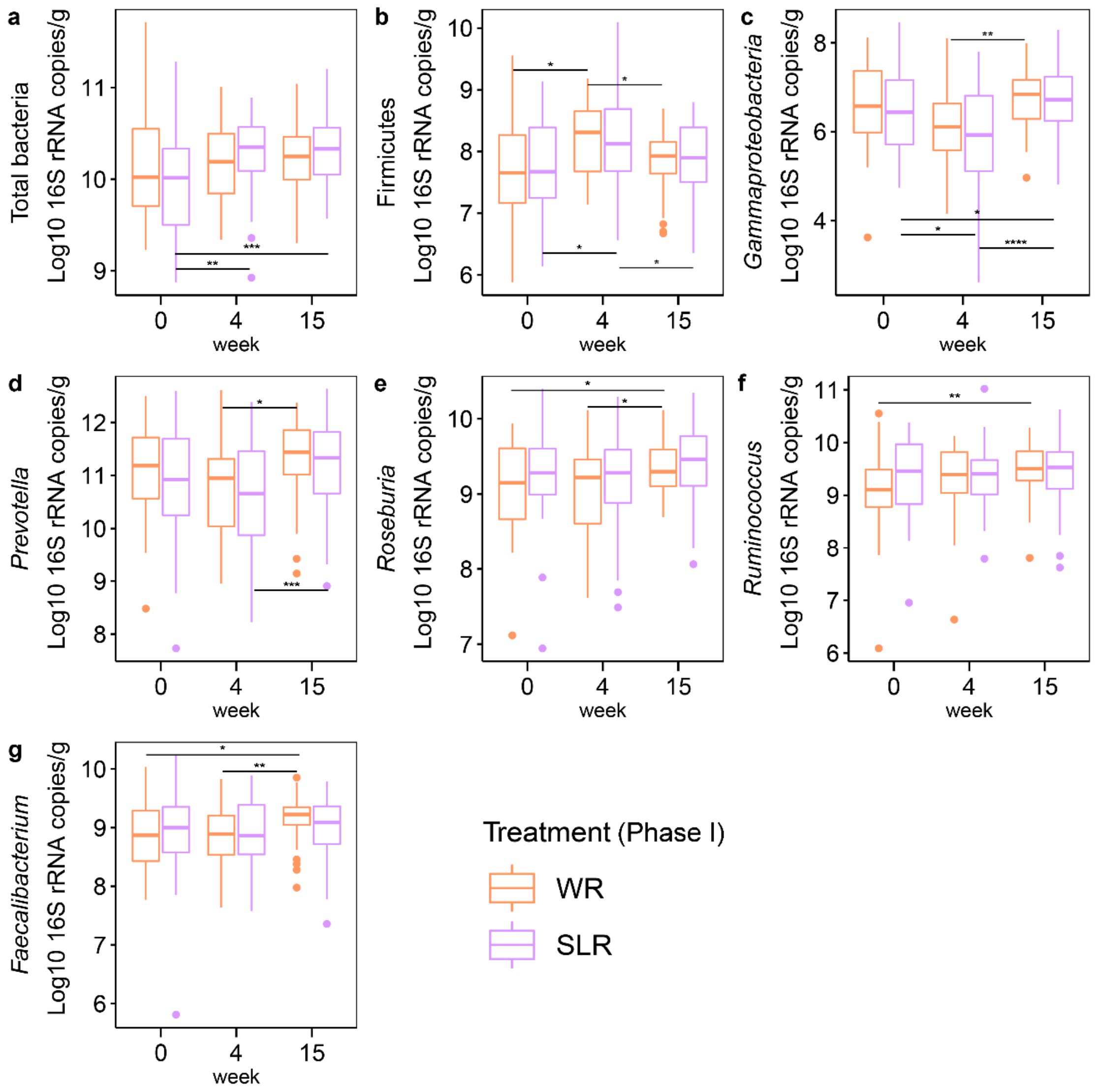

Figure S1 Boxplots represent normalized bacterial abundances based on log10 qPCR 16S rRNA copy number per gram of feces across weeks of phase I. Differences in mean absolute abundances of gut microbiota between time points of each phase (within-subjects) were determined using pairwise t-tests or Wilcoxon signed rank test with Benjamini-Hochberg (BH) *p*-value correction following the significant results of one-way repeated measures ANOVA or the Friedman test ($p < 0.05$). ****$q < 0.0001$, ***$q < 0.001$ **$q < 0.01$, *$q < 0.05$. WR, white rice (control); SLR, Sinlek rice intervention.

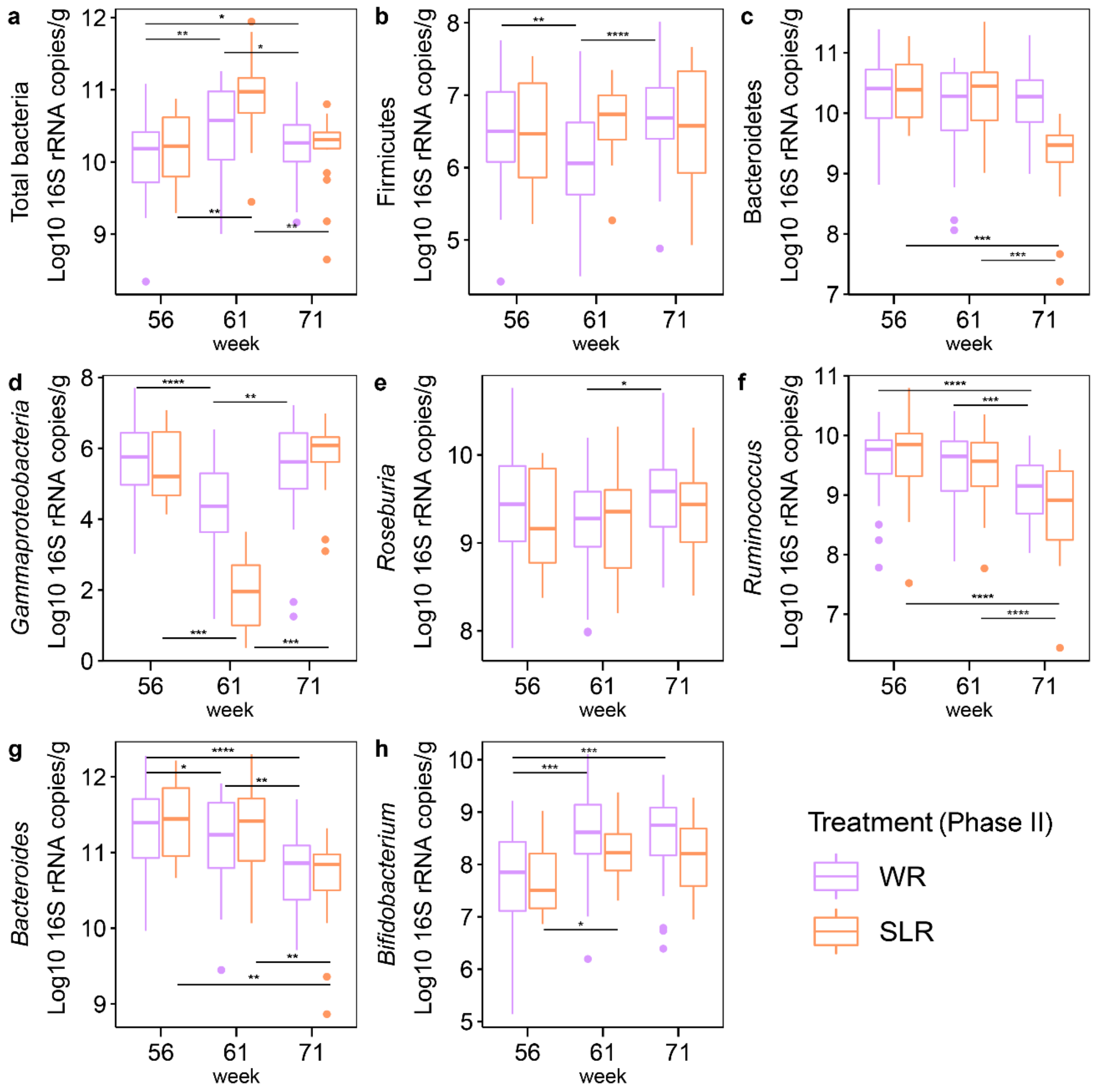

Figure S2 Boxplots represent normalized bacterial abundances based on log10 qPCR 16S rRNA copy number per gram of feces across weeks of phase II. Differences in mean absolute abundances of gut microbiota between time points of each phase (within-subjects) were determined using pairwise t-tests or Wilcoxon signed rank test with Benjamini-Hochberg (BH) p-value correction following the significant results of one-way repeated measures ANOVA or the Friedman test ($p < 0.05$). ****$q < 0.0001$, ***$q < 0.001$ **$q < 0.01$, *$q < 0.05$. WR, white rice (control); SLR, Sinlek rice intervention.

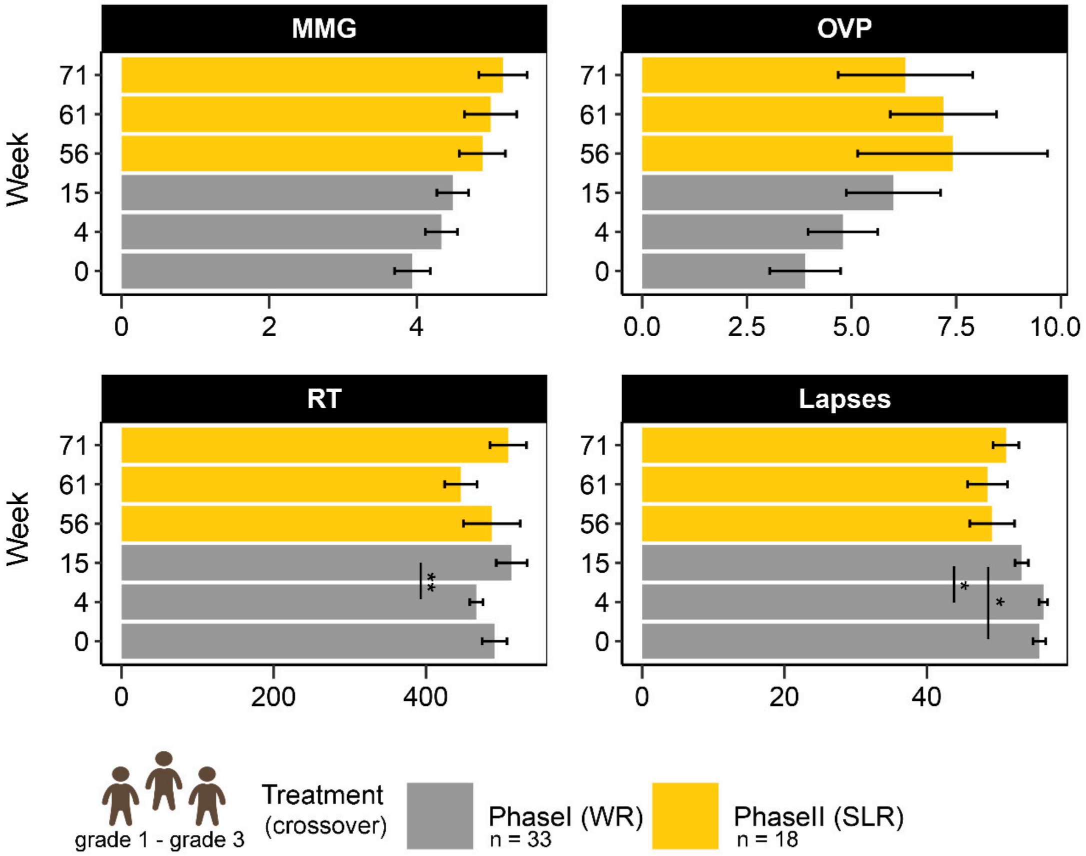

Figure S3 Barplots of cognitive performance of school-aged children in a non-randomized clinical trial. A difference in mean across weeks of each phase (within-subjects) was determined using the Wilcoxon signed rank test with Benjamini-Hochberg (BH) p-value correction following the significant results of the Friedman test ($p < 0.05$). ***$q < 0.001$ **$q < 0.01$, *$q < 0.05$. WR, white rice (control); SLR, Sinlek rice intervention; MMG = memory matching game. OVP = overall performance (%). RT = reaction times.

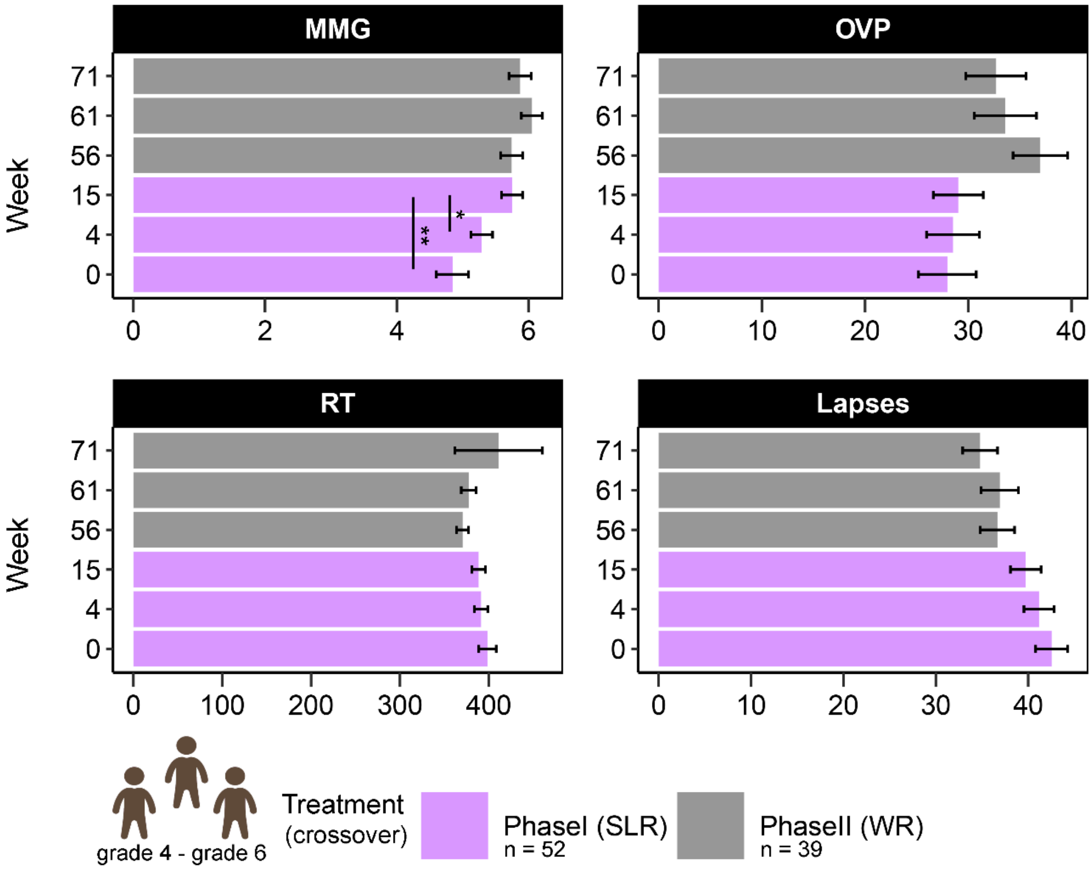

Figure S4 Barplots of cognitive performance of school-aged children in a non-randomized clinical trial. A difference in mean across weeks of each phase (within-subjects) was determined using the Wilcoxon signed rank test with Benjamini-Hochberg (BH) *p*-value correction following the significant results of the Friedman test ($p < 0.05$). ***$q < 0.001$ **$q < 0.01$, *$q < 0.05$. WR, white rice (control); SLR, Sinlek rice intervention; MMG = memory matching game. OVP = overall performance (%). RT = reaction times.

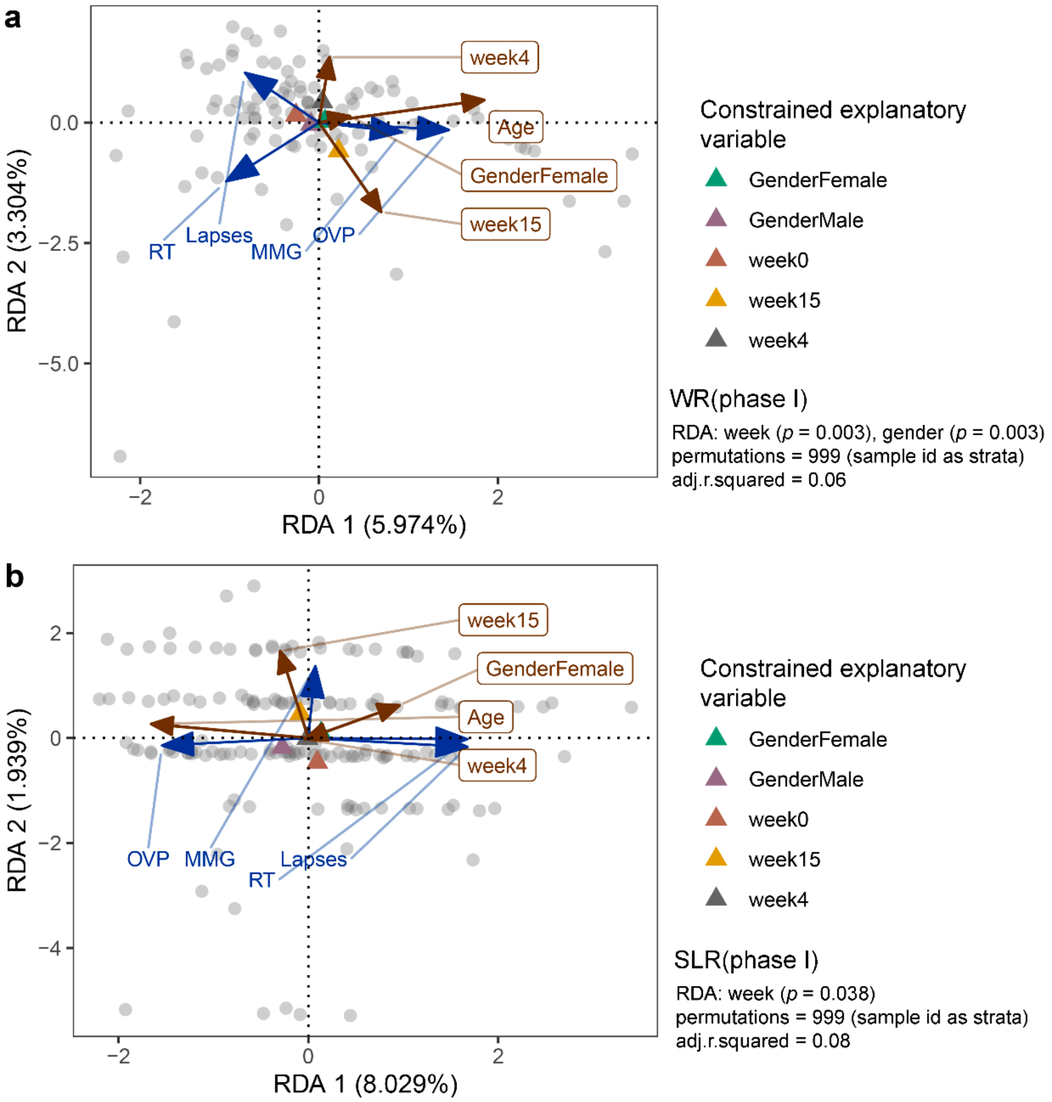

Figure S5 RDA plots for the effect of intervention on cognitive performance of school-aged children in phase I ((a) the control group (WR treatment) and (b) the intervention group (SLR treatment)). Treatment, gender, and age were used as constrained explanatory variables and the cognitive performance were used as response variables. Biplot arrows in the RDA plots represent cognitive performance (blue arrows) and constrained explanatory variables (brown arrows). A triangle is the centroids of each explanatory variable. The angles between vectors reflect their correlation. A significance of constraints was assessed using an ANOVA like permutation test. Sample ids were constrained within each treatment group and between and within variance were quantified by each week (baseline, week 4, and week 15). WR, white rice (control); SLR, Sinlek rice intervention; MMG, memory matching game; OVP, overall performance (%); RT, reaction times (millisecond); Lapses (millisecond).

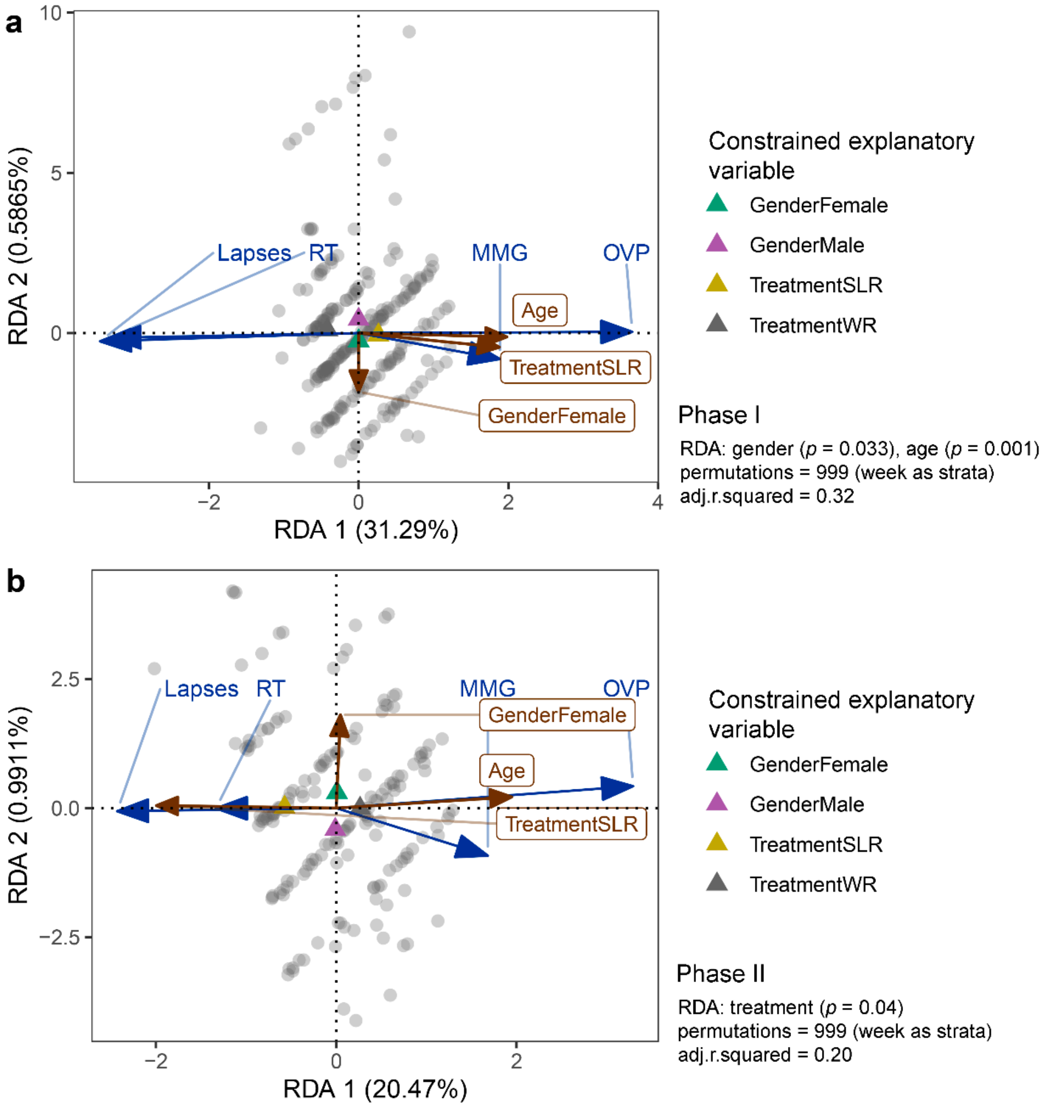

Figure S6 RDA plots for the effect of intervention on cognitive performance of school-aged children in phase I (a) and phase II (b). Treatment, gender, and age were used as constrained explanatory variables and the cognitive performance were used as response variables. Biplot arrows in the RDA plots represent cognitive performance (blue arrows) and constrained explanatory variables (brown arrows). A triangle is the centroids of each explanatory variable. The angles between vectors reflect their correlation. A significance of constraints was assessed using an ANOVA like permutation test. A week variable was constrained within each phase and between and within variance were quantified by treatment. WR, white rice (control); SLR, Sinlek rice intervention; MMG, memory matching game; OVP, overall performance (%); RT, reaction times (millisecond); Lapses (millisecond).

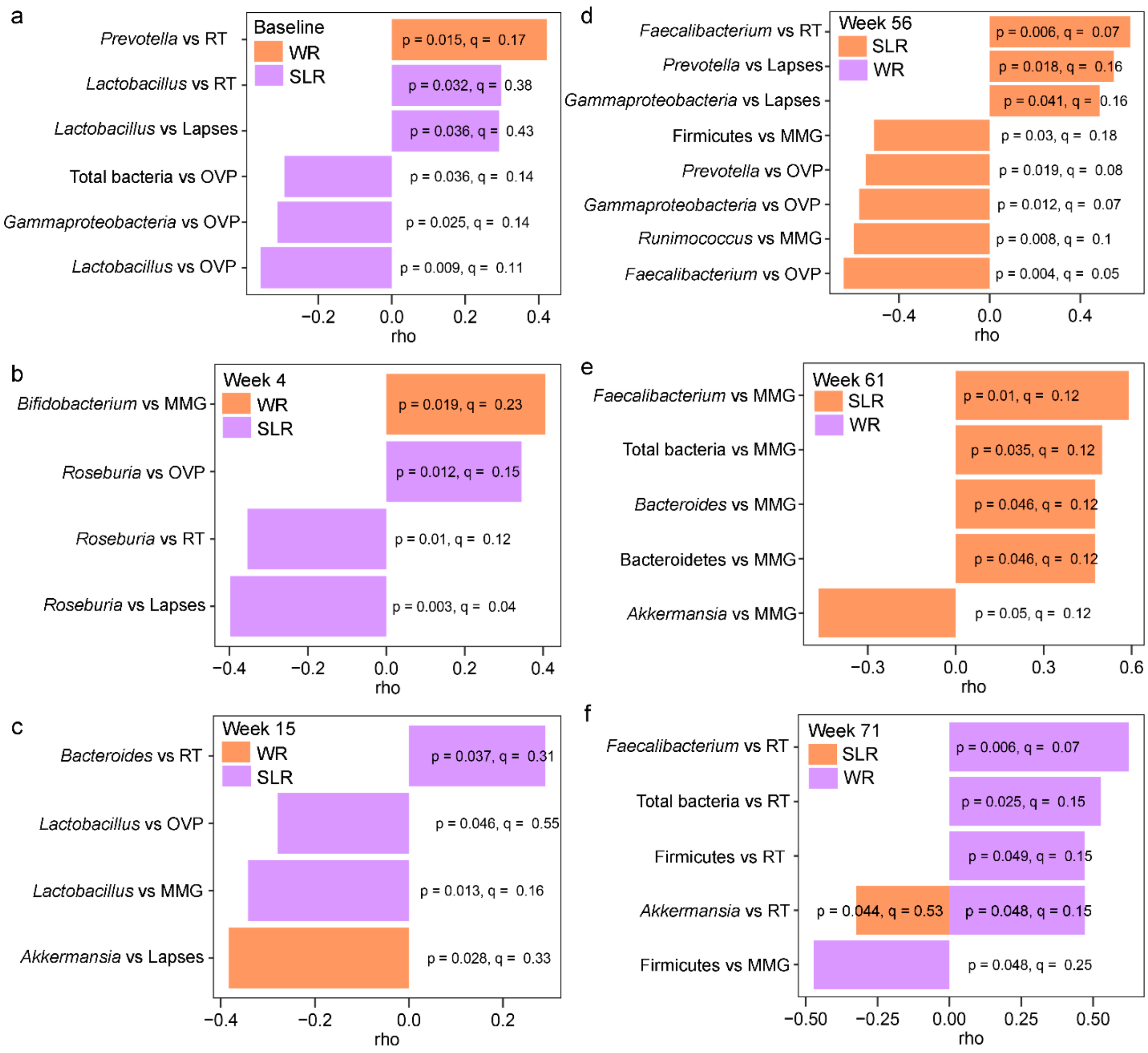

Figure S7 The relationship between gut microbiota and cognitive performance of school-aged children in a non-randomized clinical trial (phase I: a-c, phase II: d-f). Associations between gut microbiota and cognitive performance were determined by using the Spearman's rank correlation coefficient. Benjamini-Hochberg (BH) *p*-value correction was used for multiple testing adjustment (*q*-value). A *q*-value less than 0.05 is statistically significant. I. WR, white rice (control); SLR, Sinlek rice intervention; MMG, memory matching game; OVP, overall performance (%); RT, reaction times (millisecond); Lapses (millisecond).

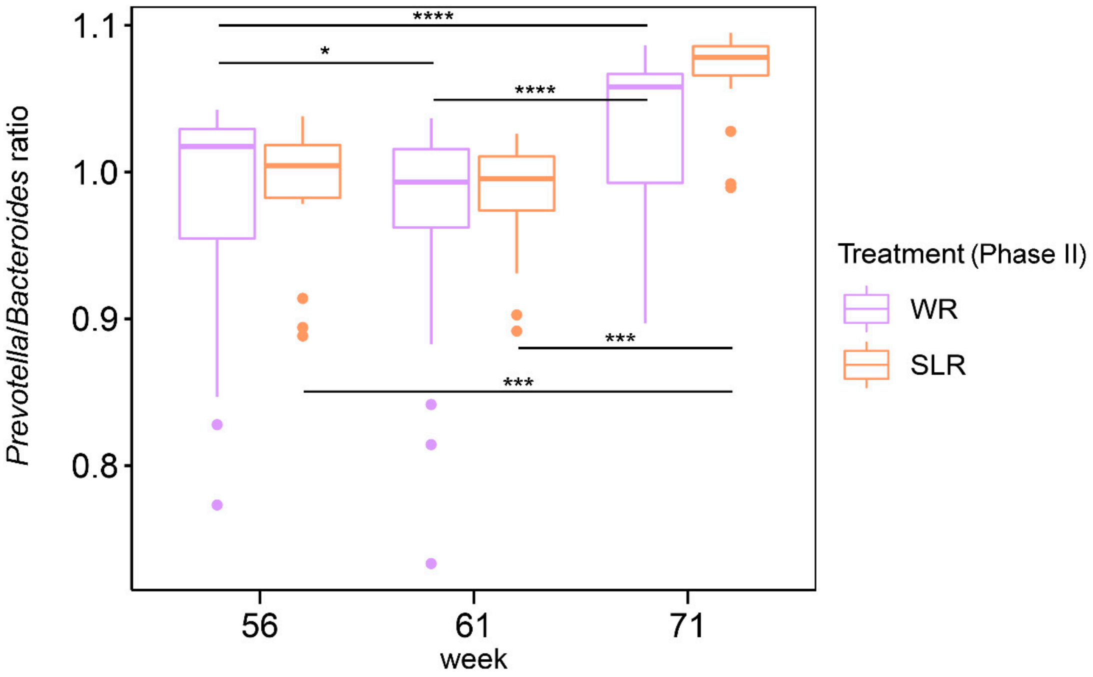

Figure S8 Boxplots represent *Prevotella*/*Bacteroides* ratio based on log10 qPCR 16S rRNA copy number per gram of feces across weeks of phase II. Differences in mean absolute abundances of gut microbiota between time points of each phase (within-subjects) were determined using the Wilcoxon signed rank test with Benjamini-Hochberg (BH) *p*-value correction following the significant results of the Friedman test ($p < 0.05$). ****$q < 0.0001$, ***$q < 0.001$ **$q < 0.01$, *$q < 0.05$. WR, white rice (control); SLR, Sinlek rice intervention.

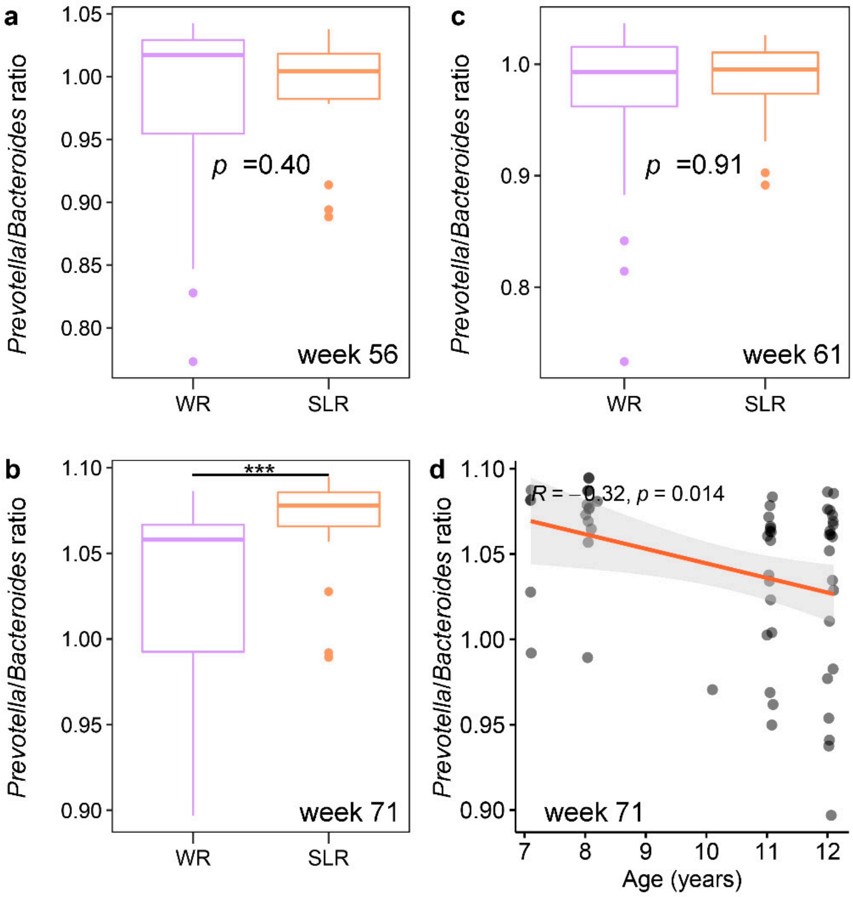

Figure S9 Boxplots represent *Prevotella*/*Bacteroides* ratio based on log10 qPCR 16S rRNA copy number per gram of feces for the control and Sinlek rice intervention in phase II. Differences in mean absolute abundances of gut microbiota between treatment groups were determined using the Wilcoxon rank sum test with Benjamini-Hochberg (BH) *p*-value correction. ***$q < 0.001$ **$q < 0.01$, *$q < 0.05$. An association between *Prevotella*/*Bacteroides* ratio and age of school-aged children was determined by using the Spearman's rank correlation coefficient. WR, white rice (control); SLR, Sinlek rice intervention.

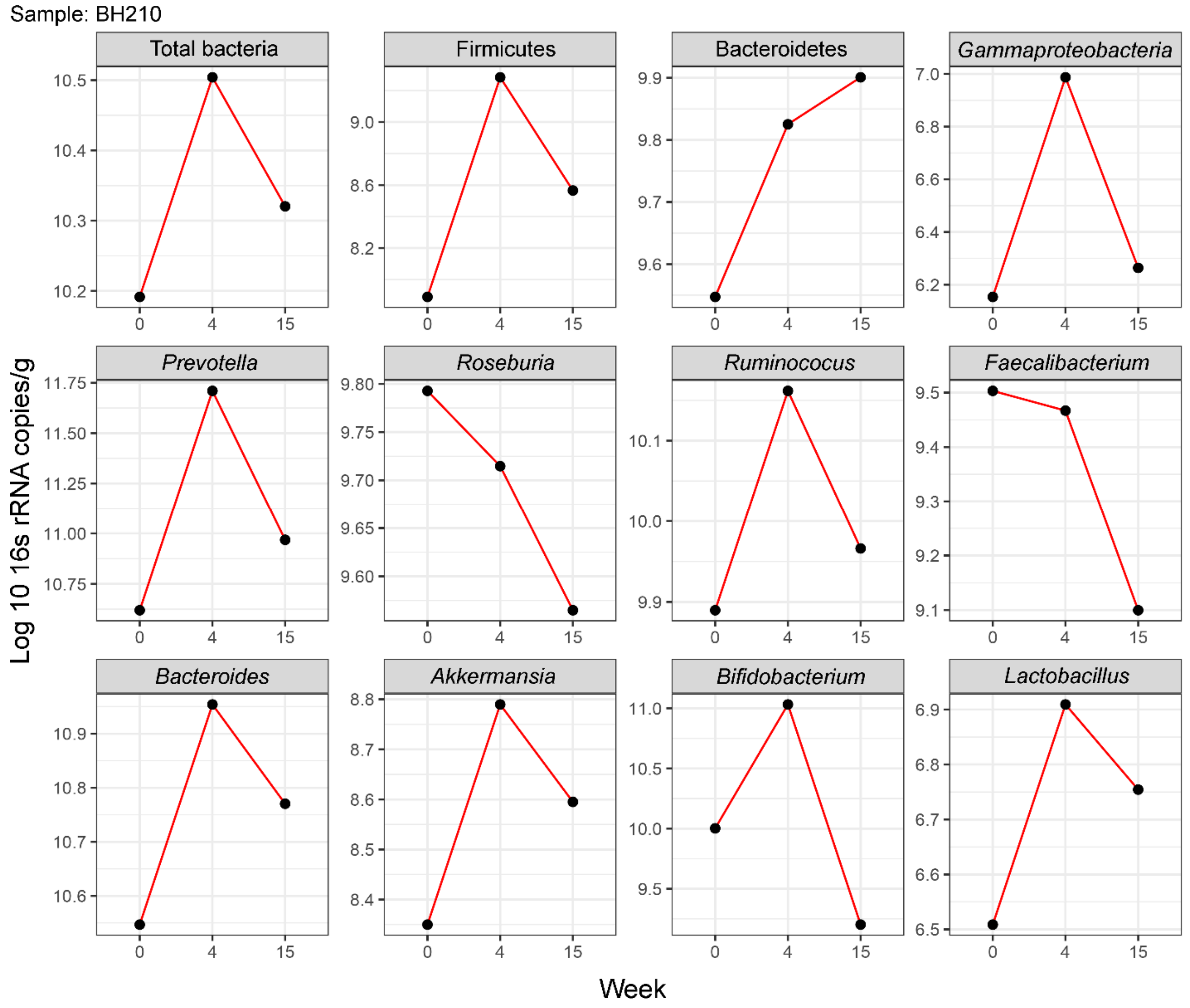

Figure S10 Boxplots of normalized bacterial abundances based on log10 qPCR 16S rRNA copy number per gram of feces of the BH210 sample across weeks of the SLR intervention (phase I). This sample was obese at baseline and then became overweight at week 4 and 15. WR, white SLR, Sinlek rice intervention.

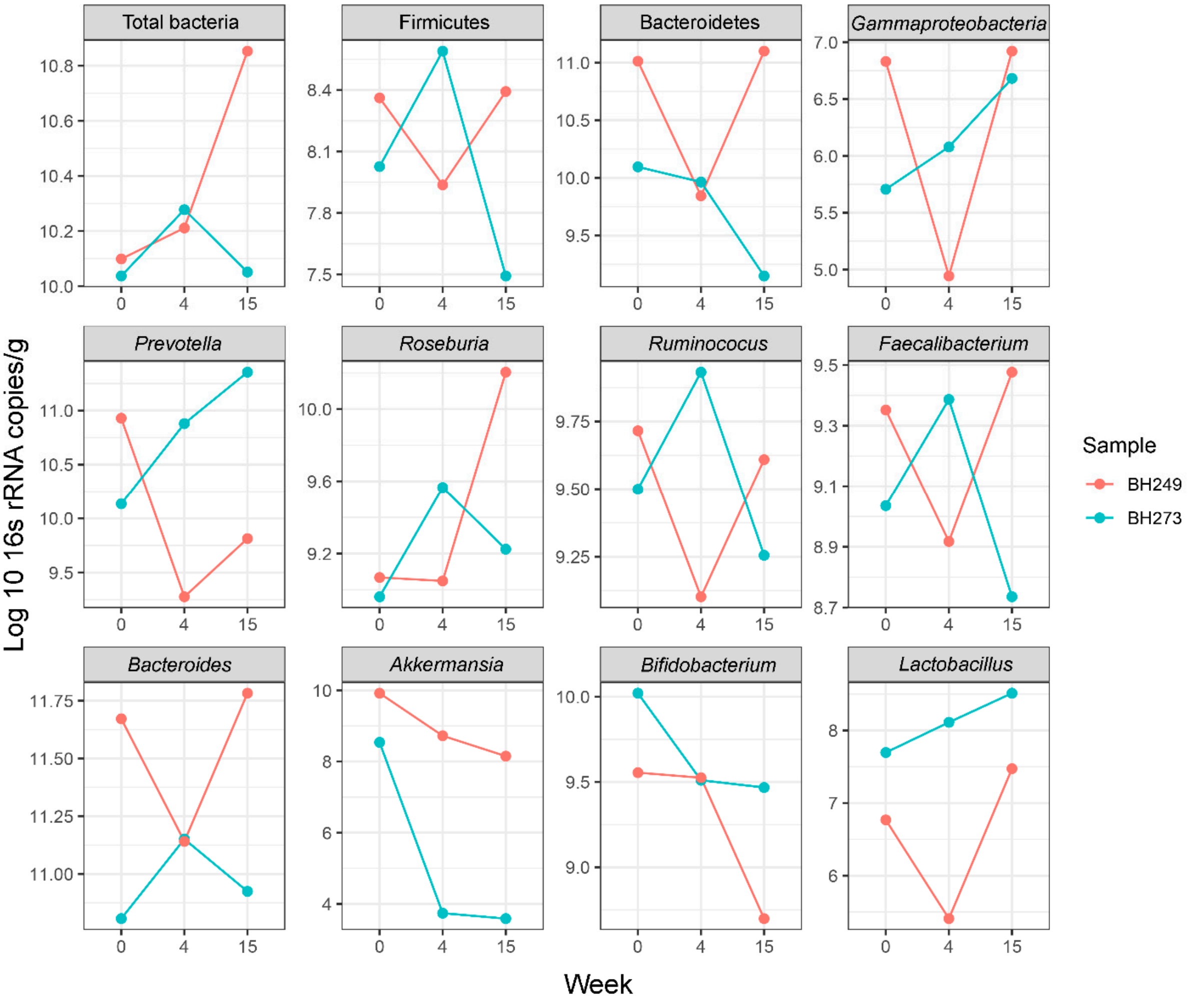

Figure S11 Boxplots of normalized bacterial abundances based on log10 qPCR 16S rRNA copy number per gram of feces of the BH2489 and BH273 samples across weeks of the SLR intervention (phase I). These samples were overweight at baseline and then became normal at week 4 and 15. SLR, Sinlek rice intervention.
