# Supplementary material for: A Non-Randomized Trial Investigating the Impact of Brown Rice Consumption on Gut Microbiota, Attention, and Short-Term Working Memory in Thai School-Aged Children
Source: Nutrients. 2022 Dec 5;14(23):5176. doi: 10.3390/nu14235176 (PMC9738792; doi:10.3390/nu14235176)
Supplement: Supplementary file 1 [file nutrients-14-05176-s001.zip › Supplementary tables.pdf]

Table S1 Primer pairs targeting bacterial 16S rRNA genes

**Target microorganism**[a]	**Primer sequence (5′—>3′)**[b,c]	**Product size (bp)**	**Ta**	**Efficiency (%)**	**Reference**
Total bacteria (Universal 180)	F: AAACTCAAAKGAATTGACGG<br>R: CTCACRRCACGAGCTGAC	180	60	91	(Bacchetti De Gregoris et al. 2011) [80]
Firmicutes (P)	F: TGAAACTYAAAGGAATTGACG<br>R: ACCATGCACCACCTGTC	200	64	87	(Bacchetti De Gregoris et al. 2011) [80]
Bacteroidetes (P)	F: CRAACAGGATTAGATACCCT<br>R: GGTAAGGTTCCTCGCGTAT	240	64	74	(Bacchetti De Gregoris et al. 2011) [80]
ɣ-*Proteobacteria* (C)	F: TCGTCAGCTCGTGTYGTGA<br>R: CGTAAGGGCCATGATG	170	53	74	(Bacchetti De Gregoris et al. 2011) [80]
Prevotella spp. (G)	F: GGTTCTGAGAGGAAGGTCCCC<br>R: TCCTGCACGCTACTTGGCTG	267	56	81	(Stevenson and Weimer 2007) [81]
Roseburia (G)	F: GCGGTRCGGCAAGTCTGA<br>R: CCTCCGACACTCTAGTMCGAC	80	55	86	(Walker et al. 2005; Ramirez-Farias et al. 2008) [82,83]
Ruminococcus (G)	F: GGCGGCYTRCTGGGCTTT<br>R: CCAGGTGGATWACTTATTGTGTTAA	156	63	68	(Ramirez-Farias et al. 2008) [83]
Faecalibacterium (G)	F: GGAGGAAGAAGGTCTTCGG<br>R: AATTCCGCCTACCTCTGCACT	247	63	79	(Ramirez-Farias et al. 2008; Wang et al. 1996) [83,84]
Bacteroides (G)	F: GAAGGTCCCCCACATTG<br>R: CGCKACTTGGCTGGTTCAG	103	64	89	(Ramirez-Farias et al. 2008; Bartosch et al. 2004) [83,85]
Akkermansia (G)	F: CAGCACGTGAAGGTGGGGAC<br>R: CCTTGCGGTTGGCTTCAGAT	349	60	91	(Collado et al. 2007) [86]
Bifidobacteria (G)	F: TCGCGTCYGGTGTGAAAG<br>R: GGTGTTCTTCCCGATATCTACA	601	64	79	(Matsuki et al. 2002; Rinttila et al. 2004) [87,88]
Lactobacillus spp. (G)	F: AGCAGTAGGGAATCTTCCA<br>R: CACCGCTACACATGGAG	341	56	84	(Walter et al. 2001; Heilig et al. 2002) [89,90]

Note: [a]P=phylum, C=class and G=genus; [b]F and R represent forward and reverse primers, respectively.
[c]Degenerate base covering: R = A/G; Y = T/C; M = A/C; W = A/T; K = T/G

References:

80. De Gregoris, T.B.; Aldred, N.; Clare, A.S.; Burgess, J.C. Improvement of phylum- and class-specific primers for real-time PCR quantification of bacterial taxa. *J. Microbiol. Methods.* **2011**, *86*, 351–356, https://doi.org/10.1016/j.mimet.2011.06.010.

81. Stevenson, D.M.; Weimer, P.J. Dominance of Prevotella and low abundance of classical ruminal bacterial species in the bovine rumen revealed by relative quantification real-time PCR. *Appl. Microbiol. Biotechnol.* **2007**, *75*, 165–174, https://doi.org/10.1007/s00253-006-0802-y.
82. Walker, A.W.; Duncan, S.H.; McWilliam Leitch, E.C; Child, M.W.; Flint, H.J. pH and Peptide Supply Can Radically Alter Bacterial Populations and Short-Chain Fatty Acid Ratios within Microbial Communities from the Human Colon. *Appl. Environ. Microbiol.* **2005**, *71*, 3692–3700, https://doi.org/10.1128/aem.71.7.3692-3700.2005.
83. Ramirez-Farias, C.; Slezak, K.; Fuller, Z.; Duncan, A.; Holtrop, G.; Louis, P. Effect of inulin on the human gut microbiota: Stimulation of Bifidobacterium adolescentis and Faecalibacterium prausnitzii. *Br. J. Nutr.* **2009**, *101*, 541–550, https://doi.org/10.1017/s0007114508019880.
84. Wang, R.F.; Cao, W.W.; Cerniglia, C.E. PCR detection and quantitation of predominant anaerobic bacteria in human and animal fecal samples. *Appl. Environ. Microbiol.* **1996**, *62*, 1242–1247, https://doi.org/10.1128/aem.62.4.1242-1247.1996.
85. Bartosch, S.; Fite, A.; Macfarlane, G.T.; McMurdo, M.E.T. Characterization of Bacterial Communities in Feces from Healthy Elderly Volunteers and Hospitalized Elderly Patients by Using Real-Time PCR and Effects of Antibiotic Treatment on the Fecal Microbiota. *Appl. Environ. Microbiol.* **2004**, *70*, 3575–3581, https://doi.org/10.1128/aem.70.6.3575-3581.2004.
86. Collado, M.C.; Derrien, M.; Isolauri, E.; de Vos, W.M.; Salminen, S. Intestinal Integrity and *Akkermansia muciniphila*, a Mucin-Degrading Member of the Intestinal Microbiota Present in Infants, Adults, and the Elderly. *Appl. Environ. Microbiol.* **2007**, *73*, 7767–7770, https://doi.org/10.1128/aem.01477-07.
87. Matsuki, T.; Watanabe, K.; Fujimoto, J.; Miyamoto, Y.; Takada, T.; Matsumoto, K.; Oyaizu, H.; Tanaka, R. Development of 16S rRNA-Gene-Targeted Group-Specific Primers for the Detection and Identification of Predominant Bacteria in Human Feces. *Appl. Environ. Microbiol.* **2002**, *68*, 5445–5451, https://doi.org/10.1128/aem.68.11.5445-5451.2002.
88. Rinttila, T.; Kassinen, A.; Malinen, E.; Krogius, L.; Palva, A. Development of an extensive set of 16S rDNA-targeted primers for quantification of pathogenic and indigenous bacteria in faecal samples by real-time PCR. *J. Appl. Microbiol.* **2004**, *97*, 1166–1177, https://doi.org/10.1111/j.1365-2672.2004.02409.x.
89. Walter, J.; Hertel, C.; Tannock, G.W.; Lis, C.M.; Munro, K.; Hammes, W.P. Detection of *Lactobacillus, Pediococcus, Leuconostoc*, and *Weissella* Species in Human Feces by Using Group-Specific PCR Primers and Denaturing Gradient Gel Electrophoresis. *Appl. Environ. Microbiol.* **2001**, *67*, 2578–2585, https://doi.org/10.1128/aem.67.6.2578-2585.2001.
90. Heilig, H.G.; Zoetendal, E.G.; Vaughan, E.E.; Marteau, P.; Akkermans, A.D.; de Vos, W.M. Molecular Diversity of *Lactobacillus* spp. and Other Lactic Acid Bacteria in the Human Intestine as Determined by Specific Amplification of 16S Ribosomal DNA. *Appl. Environ. Microbiol.* **2002**, *68*, 114–23, https://doi.org/10.1128/aem.68.1.114-123.2002.

Table S2 Demographics of school-aged children in the control and intervention groups at baseline

Demographic variables	mean (sd)		*p*-value	Test
	**WR (n = 33)**	**SLR (n = 52)**		
Age (years)	7.02 (0.71)	10.52 (0.82)	< 0.0001	Wilcoxon rank sum test
Gender (n, boys/girls)	14/19	17/35	0.49	Fisher's exact test
Weight (kg)	27.36 (11.2)	40.18 (10.99)	< 0.0001	Wilcoxon rank sum test
Height (cm)	122.11 (8.15)	144.68 (8.88)	< 0.0001	t-test
BMI (kg/m2)	17.9 (4.99)	18.97 (3.93)	0.03	Wilcoxon rank sum test
**BMI zscore (n)**				
Severe thinness	0	0		
Thinness	0	3		
Normal	20	32	0.42	Fisher's exact test
Overweight (OV)	6	11		
Obese (OB)	7	6		
**Ethnicity (n)**				
Akha	11	15		
Chinese	9	14		
Lahu	1	1	0.98	Fisher's exact test
Thai	5	9		
Thai Yai	7	13		
**Delivery mode (n)**				
Vaginal delivery	18	35	0.26	
Caesarean section	15	17		Fisher's exact test
**Feeding type (n)**				
Breastfeeding	22	47		
Formular feeding	8	3	0.02	Fisher's exact test
Mixed feeding	3	2		

A *p*-value less than 0.05 is statistically significant. Abbreviations: WR, white rice; SLR, Sinlek rice.

Table S3 Demographics of school-aged children in the control and intervention groups at week 4

Demographic variables	mean (sd)		*p*-value	Test
	WR (n = 33)	SLR (n = 52)		
Age (years)	7.08 (0.67)	10.6 (0.85)	< 0.0001	Wilcoxon rank sum test
Gender (n, boys/girls)	14/19	17/35	0.49	Fisher's exact test
Weight (kg)	27.36 (11.62)	42.54 (10.67)	< 0.0001	Wilcoxon rank sum test
Height (cm)	123.64 (8.28)	146.44 (9.12)	< 0.0001	t-test
BMI (kg/m2)	18.3 (5.01)	19.6 (3.71)	0.005	Wilcoxon rank sum test
**BMI zscore (n)**				
Severe thinness	0	0		
Thinness	0	0		
Normal	21	33	0.5	Fisher's exact test
Overweight (OV)	5	12		
Obese (OB)	7	7		
**Ethnicity (n)**				
Akha	11	15		
Chinese	9	14		
Lahu	1	1	0.98	Fisher's exact test
Thai	5	9		
Thai Yai	7	13		
**Delivery mode (n)**				
Vaginal delivery	18	35	0.26	Fisher's exact test
Caesarean section	15	17		
**Feeding type (n)**				
Breastfeeding	22	47		
Formular feeding	8	3	0.02	Fisher's exact test
Mixed feeding	3	2		

A *p*-value less than 0.05 is statistically significant. Abbreviations: WR, white rice; SLR, Sinlek rice.

Table S4 Demographics of school-aged children in the control and intervention groups at week 15

Demographic variables	mean (sd)		*p*-value	Test
	WR (n = 33)	SLR (n = 52)		
Age (years)	7.23 (0.71)	10.8 (0.85)	< 0.0001	Wilcoxon rank sum test
Gender (n, boys/girls)	14/19	17/35	0.49	Fisher's exact test
Weight (kg)	29.24 (11.35)	42.54 (10.67)	< 0.0001	Wilcoxon rank sum test
Height (cm)	123.88 (8.23)	146.65 (9.12)	< 0.0001	t-test
BMI (kg/m2)	18.6 (4.87)	19.7 (3.73)	0.01	Wilcoxon rank sum test
**BMI zscore (n)**				
Severe thinness	0	0		
Thinness	1	0		
Normal	18	33	0.54	Fisher's exact test
Overweight (OV)	7	11		
Obese (OB)	7	8		
**Ethnicity (n)**				
Akha	11	15		
Chinese	9	14		
Lahu	1	1	0.98	Fisher's exact test
Thai	5	9		
Thai Yai	7	13		
**Delivery mode (n)**				
Vaginal delivery	18	35	0.26	
Caesarean section	15	17		Fisher's exact test
**Feeding type (n)**				
Breastfeeding	22	47		
Formular feeding	8	3	0.02	Fisher's exact test
Mixed feeding	3	2		

A *p*-value less than 0.05 is statistically significant. Abbreviations: WR, white rice; SLR, Sinlek rice.

Table S5 Demographics of school-aged children in the control and intervention groups at week 56

Demographic variables	mean (sd)		*p*-value	Test
	WR (n = 39)	SLR (n = 18)		
Age (years)	11.38 (0.61)	7.66 (0.48)	< 0.0001	Wilcoxon rank sum test
Gender (n, boys/girls)	16/23	8/10	1	Fisher's exact test
Weight (kg)	43.49 (11.12)	27.89 (9.62)	< 0.0001	Wilcoxon rank sum test
Height (cm)	144.22 (8.29)	121.81 (7.48)	< 0.0001	Wilcoxon rank sum test
BMI (kg/m2)	20.7 (3.87)	18.49 (4.46)	0.01	Wilcoxon rank sum test
**BMI zscore (n)**				
Severe thinness	0	0		
Thinness	0	0		
Normal	19	10	0.55	Fisher's exact test
Overweight (OV)	14	4		
Obese (OB)	6	4		
**Ethnicity (n)**				
Akha	9	5		
Chinese	13	6		
Lahu	1	1	0.48	Fisher's exact test
Thai	9	1		
Thai Yai	7	5		
**Delivery mode (n)**				
Vaginal delivery	26	10	0.56	
Caesarean section	13	8		Fisher's exact test
**Feeding type (n)**				
Breastfeeding	37	11		
Formular feeding	1	4	0.004	Fisher's exact test
Mixed feeding	1	3		

A *p*-value less than 0.05 is statistically significant. Abbreviations: WR, white rice; SLR, Sinlek rice.

Table S6 Demographics of school-aged children in the control and intervention groups at week 61

**Demographic variables**	**mean (sd)**		***p*-value**	**Test**
	**WR (n = 39)**	**SLR (n = 18)**		
Age (years)	11.43 (0.62)	7.67 (0.47)	< 0.0001	Wilcoxon rank sum test
Gender (n, boys/girls)	16/23	8/10	1	Fisher's exact test
Weight (kg)	44 (11.36)	28.06 (9.66)	< 0.0001	Wilcoxon rank sum test
Height (cm)	146.05 (9.47)	121.81 (7.48)	< 0.0001	Wilcoxon rank sum test
BMI (kg/m2)	20.49 (3.87)	18.61 (4.44)	0.03	Wilcoxon rank sum test
**BMI zscore (n)**				
Severe thinness	1	0		
Thinness	0	0		
Normal	19	10	0.66	Fisher's exact test
Overweight (OV)	14	4		
Obese (OB)	5	4		
**Ethnicity (n)**				
Akha	9	5		
Chinese	13	6		
Lahu	1	1	0.48	Fisher's exact test
Thai	9	1		
Thai Yai	7	5		
**Delivery mode (n)**				
Vaginal delivery	26	10	0.56	
Caesarean section	13	8		Fisher's exact test
**Feeding type (n)**				
Breastfeeding	37	11		
Formular feeding	1	4	0.004	Fisher's exact test
Mixed feeding	1	3		

A *p*-value less than 0.05 is statistically significant. Abbreviations: WR, white rice; SLR, Sinlek rice.

Table S7 Demographics of school-aged children in the control and intervention groups at week 71

Demographic variables	mean (sd)		*p*-value	Test
	WR (n = 39)	SLR (n = 18)		
Age (years)	11.57 (0.55)	7.8 (0.44)	< 0.0001	Wilcoxon rank sum test
Gender (n, boys/girls)	16/23	8/10	1	Fisher's exact test
Weight (kg)	44.03 (11.6)	28.39 (9.38)	< 0.0001	Wilcoxon rank sum test
Height (cm)	146.06 (9.49)	121.86 (7.49)	< 0.0001	Wilcoxon rank sum test
BMI (kg/m2)	20.51 (4.31)	18.85 (4.37)	0.07	Wilcoxon rank sum test
**BMI zscore (n)**				
Severe thinness	1	0		
Thinness	0	0		
Normal	20	7	0.66	Fisher's exact test
Overweight (OV)	13	7		
Obese (OB)	5	4		
**Ethnicity (n)**				
Akha	9	5		
Lahu	13	6		
Chinese	1	1	0.48	Fisher's exact test
Thai	9	1		
Thai Yai	7	5		
**Delivery mode (n)**				
Vaginal delivery	26	10	0.56	
Caesarean section	13	8		Fisher's exact test
**Feeding type (n)**				
Breastfeeding	37	11		
Formular feeding	1	4	0.004	Fisher's exact test
Mixed feeding	1	3		

A *p*-value less than 0.05 is statistically significant. Abbreviations: WR, white rice; SLR, Sinlek rice.
